# Nanospray Desorption Electrospray Ionization Mass Spectrometry Imaging (nano-DESI MSI): A Tutorial Review

**DOI:** 10.1021/acsmeasuresciau.4c00028

**Published:** 2024-08-21

**Authors:** Mushfeqa Iqfath, Syeda Nazifa Wali, Sara Amer, Emerson Hernly, Julia Laskin

**Affiliations:** Department of Chemistry, Purdue University, West Lafayette, Indiana 47907, United States

**Keywords:** nanospray desorption electrospray ionization (nano-DESI), ambient ionization techniques, mass spectrometry imaging, biological imaging, shear force microscopy, tandem mass spectrometry, lipids, metabolites, matrix effects

## Abstract

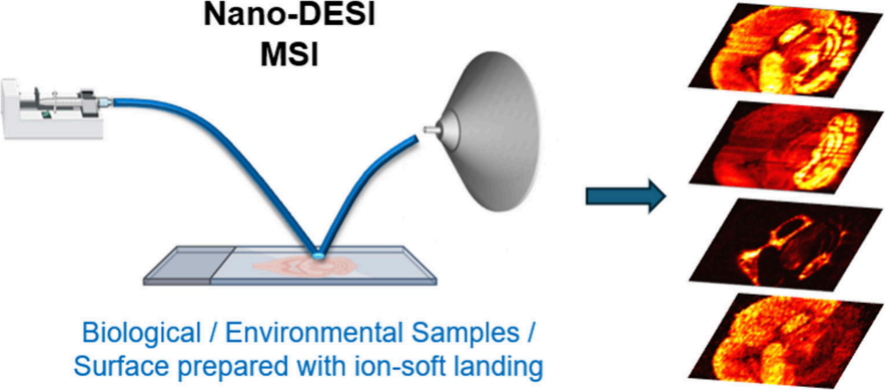

Nanospray desorption electrospray ionization (nano-DESI) is a liquid-based ambient mass spectrometry imaging (MSI) technique that enables visualization of analyte distributions in biological samples down to cellular-level spatial resolution. Since its inception, significant advancements have been made to the nano-DESI experimental platform to facilitate molecular imaging with high throughput, deep molecular coverage, and spatial resolution better than 10 μm. The molecular selectivity of nano-DESI MSI has been enhanced using new data acquisition strategies, the development of separation and online derivatization approaches for isobar separation and isomer-selective imaging, and the optimization of the working solvent composition to improve analyte extraction and ionization efficiency. Furthermore, nano-DESI MSI research has underscored the importance of matrix effects and established normalization methods for accurately measuring concentration gradients in complex biological samples. This tutorial offers a comprehensive guide to nano-DESI experiments, detailing fundamental principles and data acquisition and processing methods and discussing essential operational parameters.

## Introduction

Nanospray desorption electrospray ionization (nano-DESI) is an ambient liquid-extraction-based ionization technique for the spatially resolved analysis of molecules on substrates using mass spectrometry (MS). Since its introduction in 2010,^1^ nano-DESI has rapidly evolved and demonstrated its potential for the analysis of a broad range of analytes.^2−7^ Notably, mass spectrometry imaging (MSI) using nano-DESI (nano-DESI MSI) has been established as a powerful technique for visualizing the spatial distributions of analytes in biological systems with little or no sample pretreatment.

Nano-DESI has been used for imaging of a diverse array of biological molecules including lipids,^8−10^ steroids,^11^ eicosanoids,^12^ metabolites,^13^ glycans,^14^ proteoforms,^15−17^ native proteins and protein complexes,^18,19^ drugs, and drug metabolites^20^ in both murine^9−12,15,20−22^ and human tissues.^14^ Lipid coverage of close to 50% has been demonstrated by comparing lipids observed in nano-DESI MSI experiments to those observed using Folch extraction combined with LC-MS/MS analysis.^9^ Although imaging of neutral lipids such as triglycerides (TGs) and sterols remains challenging, nano-DESI has shown promising initial success in imaging of TGs,^10^ steroids,^11^ and cholesteryl esters.^23^ In addition to biological tissue samples, nano-DESI has been applied to the analysis of crude oil,^2^ environmental samples on substrates,^24^ living microbial colonies,^5^ and surfaces prepared using ion soft landing.^25−27^ Additionally, the gentle extraction of analytes in nano-DESI MSI has facilitated the single-cell analysis of proteoforms.^28^

In nano-DESI, analytes are desorbed from the sample into a liquid bridge formed between the sample surface and the nano-DESI probe described later in this tutorial. The desorbed analytes are transferred through the probe to a mass spectrometer inlet either by self-aspiration or vacuum-assisted flow and ions are generated by electrospray-like ionization.^1,29^ In MSI experiments, the sample is scanned continuously under the nano-DESI probe while mass spectra are acquired as discrete events. This distinguishes nano-DESI MSI from both laser- and ion beam-based sampling of analytes, which generate physically separated packets of ions.

Like other ambient ionization techniques, nano-DESI requires no special sample pretreatment, making it more accessible to laboratories that are not equipped for matrix application used in matrix-assisted laser desorption ionization (MALDI) and some other LDI-based MSI techniques. Additionally, the absence of matrix peaks in the low *m*/*z* range facilitates imaging of metabolites.^3,5,8^ Although lipid imaging using MALDI and nano-DESI generates comparable results, nano-DESI MSI has successfully mapped certain drug molecules and their metabolites that were not observed using MALDI.^20^ Furthermore, multiple charging in nano-DESI is advantageous for imaging and identification of proteins.^17^ The flexibility in solvent composition contributes to the quantitative capabilities of nano-DESI MSI.^30^ Notably, adding standards to the working solvent helps compensate for signal suppression during ionization, allowing for relative quantification in each experiment without increasing the complexity of the experimental setup.^31^ The limitations of nano-DESI stem from the necessity for the analyte of interest to be soluble in the working solvent. This presents a challenge for detecting poorly soluble analytes or those strongly bound to other biomolecules in the complex biological sample. Additionally, analytes with low electrospray ionization efficiency may be difficult to detect in nano-DESI MSI experiments. Unlike MALDI MSI, nano-DESI has not yet been commercialized and requires the development of a custom-built source. Several protocols^8,29^ and tutorials^32^ provide guidance for the construction a nano-DESI setup and implementing it on commercial mass spectrometers.

Advancements to nano-DESI MSI have been well-documented, including increased sensitivity achieved through the introduction of pneumatic assistance to the probe using a nebulizer instead of a conventional self-aspirating secondary probe.^13^ Furthermore, significant efforts have been dedicated to enhancing the molecular selectivity of the technique. For example, the implementation of nano-DESI on a Fourier transform ion cyclotron resonance (FT-ICR) MS has been used to attain an improved mass resolution and sub-ppm accuracy necessary to obtain isotopic fine structure information.^33^ Meanwhile, coupling of nano-DESI MSI with ion mobility spectrometry (IMS) along with the development of MS^2^, MS^3^, and MS^4^ imaging approaches have been used for separating isomeric and closely spaced isobaric species.^34−39^ Isomer separation has also been demonstrated using online derivatization approaches, opening new directions in understanding the role of isomeric lipids and metabolites in biological systems.^36^

Optimizing the performance of the nano-DESI source requires careful adjustment of several parameters including solvent composition and voltage gradient at the MS inlet along with the position of the nano-DESI probe relative to the sample and instrument inlet. Additionally, the spatial resolution of nano-DESI MSI experiments is influenced by the geometry of the nano-DESI probe, the solvent flow rate, the properties of the sample surface, and the precise control of the distance between the sample and the probe. A three-point plane calibration method has been used to automatically adjust for the tilt of the sample surface providing a stable operation of the nano-DESI probe in MSI experiments performed with a spatial resolution in the range of 20–200 μm.^40^ Meanwhile, a more precise method has been developed for imaging of tissue sections with higher spatial resolution. In this method, a shear force probe^41^ is used to control the distance between the sample and a high-resolution nano-DESI probe that generates a small liquid bridge on the sample surface. This approach has enabled imaging with high spatial resolution of better than 10 μm as described later in the text.^42^

These capabilities of nano-DESI MSI, combined with the flexibility in selecting solvent composition for specific applications have established it as a powerful technique for ambient ionization-based MSI with enhanced resolution and throughput. This article offers a comprehensive tutorial of nano-DESI MSI covering various aspects including the setup of different types of nano-DESI probes, spatial resolution, experimental throughput, and different modes of data acquisition and data processing. Additionally, it addresses solvent selection and the impact of matrix effects on the results of nano-DESI MSI experiments.

## Nano-DESI Probes

**Figure 1:**
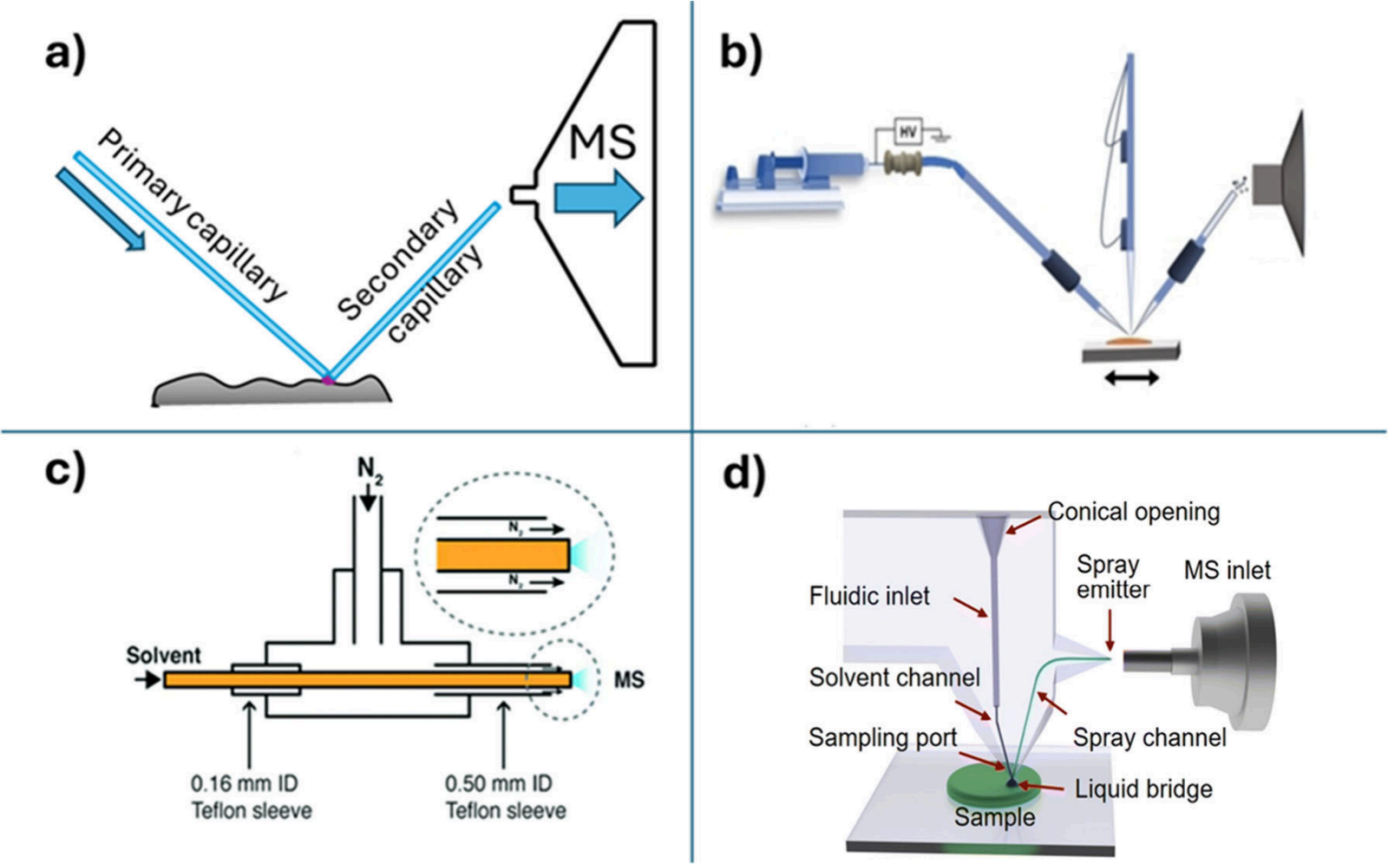
Nano-DESI probe setups. (a) Nano-DESI configuration of a moderate resolution nano-DESI probe. Adapted with permission from ref (20). Copyright 2022 Elsevier. (b) Schematic showing a high-resolution nano-DESI setup. Adapted with permission from ref (17). Copyright 2022 Wiley-VCH GmbH. (c) Schematic of a pneumatically assisted probe. Reproduced with permission from ref (13). Copyright 2017 The Royal Society of Chemistry. (d) Schematic of an MFP fabricated using femtosecond selective laser-assisted etching. Reprinted with permission under a Creative Commons Attribution 3.0 Unported License from ref (43). Copyright 2023 The Royal Society of Chemistry.

The design of nano-DESI probes has been described in detail in our previous publications^29,32,43^ and will be briefly summarized here. A majority of nano-DESI MSI experiments reported so far utilized a probe composed of two glass capillaries. A schematic drawing of the nano-DESI probe for imaging with moderate spatial resolution is shown in Figure 1a. In this probe, a primary capillary is connected to the syringe filled with the working solvent. The syringe is mounted on a syringe pump that propels the solvent through the primary capillary. Meanwhile, a secondary capillary (or nanospray capillary), which is quite short (∼2 cm), is mounted near the MS inlet. The capillaries are arranged at a ∼ 90° angle and properly positioned relative to one another and to the instrument inlet. Depending on the type of a mass spectrometer, a high voltage is applied either to the syringe needle or to the instrument inlet to generate a voltage gradient for ionization. The working solvent is delivered from the primary to the secondary capillary thereby generating a liquid bridge at the junction between the two capillaries. When the liquid bridge is brought in contact with a sample, it forms a meniscus on the surface. Molecules extracted from the sample into the liquid bridge are transferred through the secondary capillary to the MS inlet where they are ionized by electrospray ionization.^8^

For imaging with high spatial resolution down to 10 μm, the capillaries are finely pulled to obtain a smaller diameter tip, which helps generate a small liquid bridge.^29^ The high-resolution nano-DESI probe is depicted in Figure 1b. In this setup, a third pulled capillary is installed close to the nano-DESI probe and operated as a shear force probe, which maintains a constant distance between the nano-DESI probe and sample during the MSI experiment.^41^ The operation of the shear force probe is described later in the text. The constant distance mode imaging approach enables both imaging of samples with complex topography such as microbial colonies^41^ and imaging of biological tissues with high spatial resolution.^42^

Because there is no active pumping of the working solvent through the secondary capillary, the nano-DESI probe is typically positioned close to the MS inlet to use vacuum pumping from the instrument to assist solvent flow through the secondary capillary. To eliminate the dependence of nano-DESI MSI on the vacuum suction from the MS inlet, Duncan et al. have developed a pneumatically assisted nano-DESI probe illustrated in Figure 1c.^13^ In this probe, a nebulizer gas is introduced through a PEEK tee to generate a gas-assisted flow of the solvent through the secondary capillary. A tapered pneumatically assisted nano-DESI probe has been used for global metabolomics of individual INS-1 cells.^44^ In another study, a similar pneumatically assisted probe was used in combination with high-field asymmetric ion mobility spectrometry to enhance protein coverage in nano-DESI MSI.^45^

The latest advancement in the nano-DESI probe technology is the development of microfluidic nano-DESI probes (MFPs).^43,46,47^ A photograph of one of the MFPs is shown in Figure 1d. MFPs use two microfluidic channels connected at the sampling port to generate the dynamic liquid bridge on the sample surface. Similar to the capillary-based nano-DESI probe, analytes extracted into the liquid bridge are ionized at MS inlet. The incorporation of the solvent delivery system and sampling port into a single device substantially simplifies the experimental setup and reduces the time required to position the nano-DESI probe in front of the MS inlet. Several methods of fabrication of MFPs have been reported.^46−48^ The traditional approach relies on photolithography-based fabrication of the channels and high-temperature bonding performed in the clean room. Manual grinding and polishing of the MFP is required for properly shaping the sampling port and the emitter tip, which generate a stable liquid bridge on the sample surface and stable electrospray, respectively.^47^ Recently, femtosecond selective laser-assisted etching has been used to fabricate several designs of MFPs by drawing channels inside the glass substrate and removing the modified glass material using chemical etching.^43^ An example of a monolithic probe fabricated using this approach is shown in Figure 1d.^43^ Femtosecond selective laser-assisted etching eliminates the need for a clean room and allows for the simultaneous fabrication of multiple devices with little to no polishing prior to their operation. Furthermore, it facilitates rapid prototyping of the MFPs. For example, a much simpler design of the V-shaped probe, in which only the sampling port of the MFP is fabricated using femtosecond selective laser-assisted etching, was readily optimized through only a few iterations.^43^ The robustness of the probe enables constant distant mode imaging by using the three-point plane calibration. Although the shear-force probe feedback method can be implemented by attaching the piezo plates to the microfluidic probe, this capability has not been described in literature.

## Constant Distance Mode Nano-DESI MSI

In nano-DESI MSI, the probe, once assembled and positioned at an optimized angle and distance from the MS inlet, remains stationary throughout the experiment. The positioning of the probe is optimized to achieve a constant and stable liquid bridge and stable electrospray signal. The flow rate of the solvent through the primary capillary, usually kept between 0.4 and 1.0 μL/min, is optimized based on the chemical composition of the solvent, hydrophobicity of the sample surface, positioning of the probe, and the vacuum of the instrument to ensure a stable liquid bridge. The inner diameter of the capillary is also a contributing factor to the optimal flow rate. Based on our experience, capillaries with larger inner diameters require a higher flow rate for the formation of a stable liquid bridge. The sample mounted on a glass slide is placed on a motorized XYZ-stage and the stage is scanned at a constant speed below the probes during the imaging experiment. The XYZ-stage is controlled using a custom-designed LabVIEW program, which synchronizes the sample stage with the mass spectrometer, streamlining the process of data acquisition.^40^ A rectangular area on the sample surface is defined for imaging with start and end positions in X and Y coordinates. The sample is scanned along the *X*-direction at a user-defined scan speed, which is in the range of 10–500 μm/s. Sequential lines are acquired by moving the stage back to the X-start position and incrementing by one step size in the *Y*-direction, effectively covering the defined region. The pixel size along the X direction is defined by the scan speed at which the stage is moving and the acquisition rate of a mass spectrometer. For example, if the scan speed is 20 μm/s and the acquisition rate is 1 Hz, then the stage will move 20 μm during the acquisition of one spectrum corresponding to one pixel in the image. Consequently, the size of the pixel in the *X*-direction will be 20 μm. In the Y direction, the pixel size is defined by the distance between two consecutive lines. It is important to note that the pixel size is different from the spatial resolution, which depends primarily on the size of the liquid bridge, discussed in a separate section later in this tutorial. In the *Z*-direction, two important parameters that need to be specified to begin the scan are *Z-start* and *Z-out* positions. *Z-start* is the height from which the stage will start moving toward the nano-DESI probe at the beginning of each line scan and *Z-out* is the position at which the stage will move back before starting the next line. The nano-DESI probe is positioned using high-resolution micromanipulators and monitored using two Dino-Lite digital microscopes. A detailed description of the assembly of both moderate- and high-resolution capillary-based nano-DESI probes is provided in previously published protocols.^8,29^

**Figure 2:**
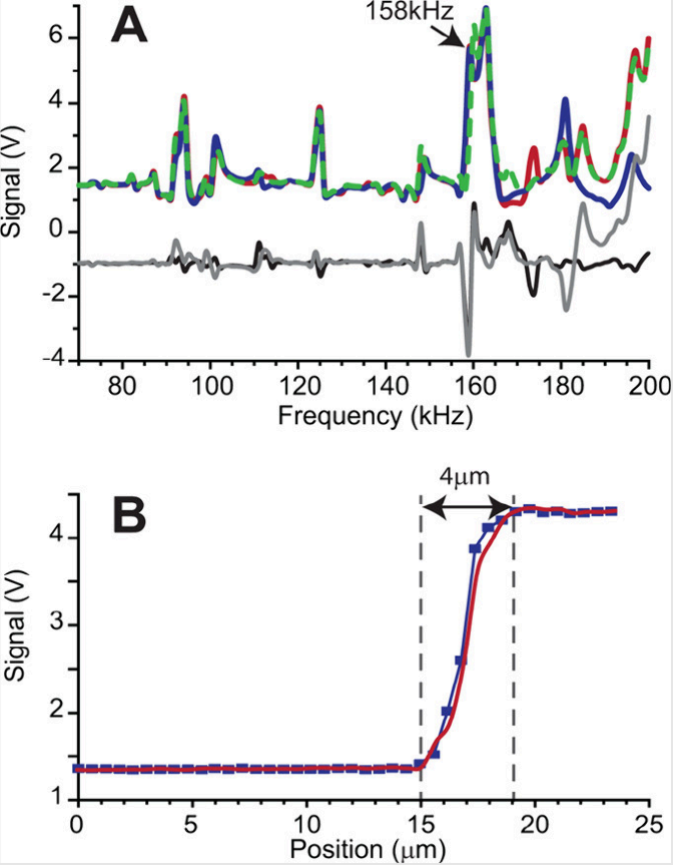
Excitation spectra representing the amplitude of the shear-force probe vibration as a function of the agitation frequency (70–200 kHz). (A) Excitation spectra acquired with the probe kept in air (dashed green trace), positioned on the agar surface (blue trace), and positioned on top of a 1 day old (1D) Bacillus ATCC 49760 colony (red trace). Black trace represents the difference spectrum between air and agar, while gray trace is a difference spectrum between air and colony. The arrow indicates the resonant frequency selected for imaging experiments. At this frequency, signals obtained from both the agar and colony have the same amplitude that is substantially different from the signal amplitude obtained in air. (B) Amplitude of the shear-force probe vibration at a frequency of 158 kHz as a function of the distance between the sample and the probe. Almost identical sharp approach curves were obtained on agar (blue trace) and colony (red trace). Reproduced with permission from ref (41). Copyright 2016 American Chemical Society.

The stability and size of the liquid bridge may be affected by the topography of the sample surface, especially for imaging using high-resolution probes. Constant-distance mode approaches have been developed to maintain the constant size of the liquid bridge and thereby eliminate the effect of surface topography on MS signal. The simplest approach based on the three-point plane calibration mentioned earlier has been used for constant distance mode imaging with a spatial resolution down to 20 μm.^40^ To achieve higher resolution nano-DESI MSI, a more precise control over Z-positioning of the sample is necessary. Specifically, for imaging with 10 μm spatial resolution, the distance between the nano-DESI probe and sample surface must be controlled to within ∼1 μm. Such control has been achieved by incorporating a shear force probe into the nano-DESI MSI system.^42^ The shear force probe is a pulled glass capillary with two piezoelectric plates attached to it. The upper plate (agitation piezo) induces probe oscillation via a function generator and the lower plate (detection piezo) positioned closer to the sample is connected to a lock-in-amplifier that detects the amplitude of probe’s vibration at a chosen frequency. This amplitude of probe’s vibration in the air will be significantly different than the amplitude on the surface. To enable constant-distance mode imaging, a specific frequency is selected that corresponds to the maximum difference in the amplitude of vibration between the air and the sample surface. This amplitude difference is maintained at a constant level through a computer-controlled closed-feedback loop that regulates the position of the stage in the *Z*-direction. Details about the shear force probe technology used in nano-DESI experiments are provided in the previous publication.^41^Figure 2A compares the excitation spectra of the shear force probe acquired in air and on the surface of a living *Bacillus subtilis* ATCC 49760 colony on an agar plate and shows difference spectra acquired at two distinct locations. The difference spectra indicate that the frequency of 158 kHz is well-suited for imaging of both the sample surface and areas outside of the sample on the agar plate. Two approach curves obtained by fixing this frequency and recording the amplitude of the shear force probe’s vibration as a function of the distance between the sample (colony or agar) and the probe are shown in Figure 2B.^41^ Based on the approach curves, the amplitude of ∼80% relative to the value at this frequency obtained when the shear force probe is in the air is selected for imaging. The XYZ stage is programmed to automatically adjust the Z-position of the sample based on the measured amplitude of the shear force probe vibration at the selected frequency throughout the MSI experiment.

## Data Acquisition

The nano-DESI source has been combined with different commercial MS instruments. Details of the hardware and software modifications associated with the implementation of a custom-designed nano-DESI platform with different mass spectrometers are provided in our recent article.^32^ Data acquisition software of the MS is used to control data acquisition in an imaging experiment. Important MS parameters selected in each experiment include the ionization mode (positive or negative), high voltage for electrospray ionization, capillary temperature, and *m*/*z* range. Multiple acquisition modes have been employed in nano-DESI MSI experiments. Although a majority of studies have relied on the acquisition of a full MS spectrum in each pixel of the image, selected ion monitoring (SIM),^12^ tandem mass spectrometry (MS/MS),^36,49^ and ion mobility spectrometry (IMS)^37,38^ have all been employed to enhance the chemical specificity and sensitivity of nano-DESI MSI experiments.

### Full MS Mode

In the full MS mode, a wide range of *m*/*z* is selected and configured within the software specific to the operational instrument. Each mass spectrum acquired during the imaging experiment represents signals of numerous *m*/*z* in one pixel of the image.^23^ The parameters that define how many spectra/scan cycles are acquired per line vary from instrument to instrument. For example, Thermo Fisher instruments (i.e., Orbitrap or linear ion trap), when operated with the automatic gain control (AGC),^50^ generate lines with a variable number of mass spectra. AGC controls the time during which a fixed number of ions is accumulated for the analysis, which may vary from spectrum to spectrum. Meanwhile, for other instruments (for example IM-QTOF, QqQ), the acquisition rate is fixed throughout the imaging experiment, typically generating pixels with uniform size and minimal variation in the number of pixels among the lines.

Once all the parameters are configured, data acquisition starts by the use of an external trigger commonly referred to as contact closure.^51^ In our laboratory, this external control is triggered by a DC pulse, that is generated by using a multifunctional I/O device (model USB-6009) from National Instruments (Austin, TX, USA). The device is interfaced with the custom-designed LabVIEW program that controls the nano-DESI source.^40,32^ It is important to note that for all the MS instruments, there is a delay between acquiring mass spectra and saving the data file on the hard drive. Therefore, for each line scan, a slightly shorter acquisition time must be selected in the mass spectrometer data acquisition software than the time required to complete one line scan across the tissue as specified by the LabVIEW program. Although the time it takes the stage to move to the beginning of the next line is short (∼1–2 s), a delay time is incorporated into the workflow to allow for data to be saved onto the hard drive. It is important to ensure that the total acquisition time of the mass spectrometer method is shorter than the time required for the stage to complete a line scan and move to the next line. This prevents communication issues with a mass spectrometer.

Usually, after each imaging experiment, on-tissue MS/MS data are acquired using either a targeted *m*/*z* list or an automated data-dependent MS/MS experiment. Species observed in MSI experiments are identified based on the accurate *m*/*z* and MS/MS data.

### Selected Ion Monitoring (SIM) Mode

**Figure 3:**
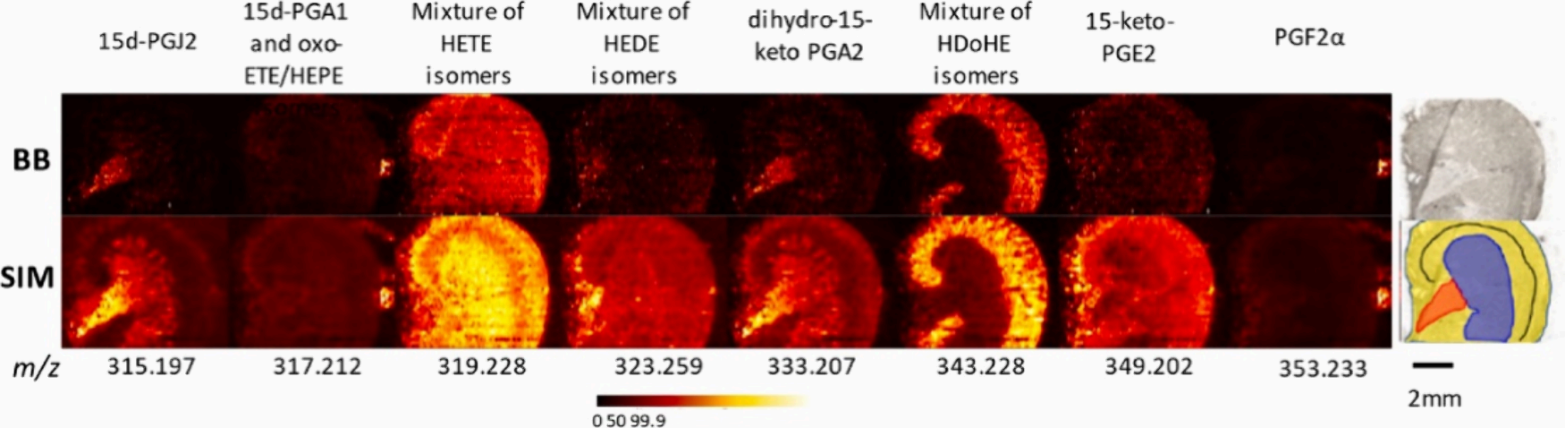
Comparison of ion images of eight low-abundant species acquired from mouse kidney tissue in broadband (top row) and SIM modes (bottom row). From left to right: 15d-PGJ2 (*m*/*z* 315.196), 15d-PGA1 (*m*/*z* 317.212), mixture 5-, 11-, 12-, 15-HETE isomers (*m*/*z* 319.227), mixture of 11- and 15-HEDE isomers (*m*/*z* 323.258), dihydro-keto PGA2 (*m*/*z* 333.206), HDoHE isomers (*m*/*z* 343.227), 15-keto-PGE2 (*m*/*z* 349.201), and PGF2α (*m*/*z* 353.233). Scale bar represents 2 mm, color bar represents 0–99.9% intensity scale. An optical image of the tissue section and the same image with color coded regions are shown in the right. Color coding highlights the inner medulla (red), medulla (purple), cortex (yellow), and corticomedullary junction (black). Reproduced with permission from ref (12). Copyright 2023 Elsevier.

In SIM mode imaging, one or multiple narrow ranges of *m*/*z* are chosen instead of a broad range.^12,22^ SIM mode is useful for the sensitive and targeted imaging of analytes on Thermo instruments. SIM mode helps with identifying and quantifying analytes with lower ionization efficiency and/or low concentration in tissue. Selective accumulation of ions in a narrow *m*/*z* window performed in SIM mode results in a substantial increase in the signal-to-noise (S/N) and a corresponding improvement in the quality of ion images. For example, SIM mode coupled with nano-DESI MSI has been utilized for the quantitative imaging of diclofenac and its low-abundance metabolites in mouse kidney and liver tissue.^20^ This study compared imaging in SIM and full MS modes. The observed improvement in the S/N ratio in SIM mode enabled accurate determination of distinct localization patterns of the drug and metabolites in the tissue. Another study utilized a series of adjacent multiple SIM (mSIM) windows to perform nano-DESI MSI of low-abundance eicosanoids in kidney tissue.^12^Figure 3 shows a comparison between broadband (BB) and SIM acquisition modes demonstrating the improvement in the quality of ion images obtained for several low-abundance species in the mSIM mode facilitated by 1–2 orders of magnitude improvement in S/N.^12^ In another study, quantitative imaging of prostaglandins was performed in SIM mode by adding a deuterated standard to the solvent and normalizing the signals of endogenous species to that of the standard.^52^

### MS/MS Mode

**Figure 4:**
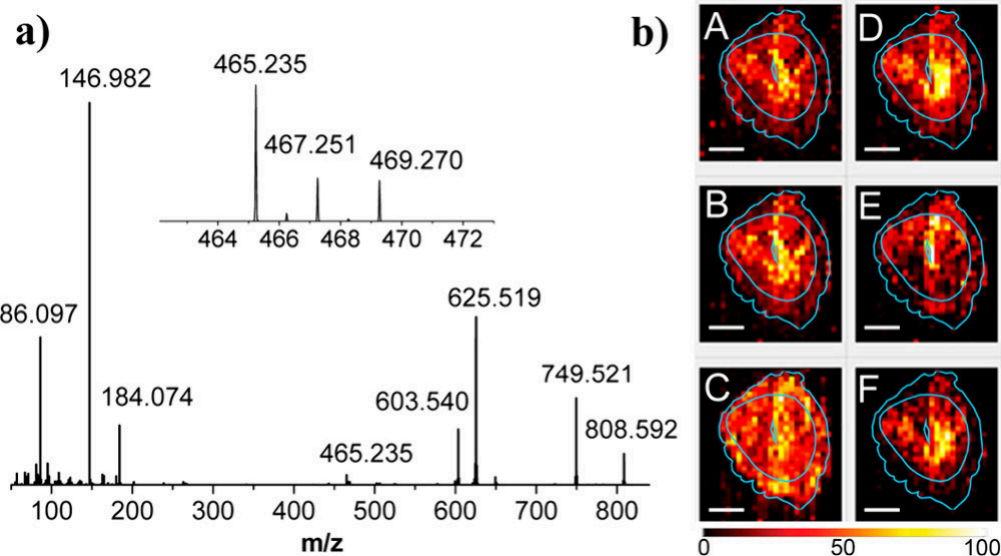
(a) Average MS/MS spectrum of *m*/*z* 808 ± 1. (b) Ion images of (A) precursor ion at *m*/*z* 808.592 and selected fragment ions: B, *m*/*z* 749.521 (NL 59.07 from [PC 36:2 + Na]^+^); C, *m*/*z* 184.074 (characteristic fragment of [PC 38:5 + H]^+^); D, *m*/*z* 465.235 (loss of FA 18:0 from [PC 36:2 + Na]^+^); E, *m*/*z* 467.251 (loss of FA 18:1 from [PC 36:2 + Na]^+^); and F, *m*/*z* 469.27 (loss of FA 18:2 from [PC 36:2 + Na]^+^). Lateral scale bar is 1 mm. Intensity scale bar ranges from 0 (black) to 100% (light yellow) signal intensity of an individual peak. Reproduced with permission from ref (34). Copyright 2013 American Chemical Society.

MS/MS-MSI is a powerful approach for separating both isobaric and isomeric species extracted from the sample by plotting the abundance of their diagnostic fragments in each pixel of the image.^34,36,49,53^ In nano-DESI MS/MS-MSI experiments, an inclusion list containing the *m*/*z* of the targeted precursors is created by the user. A mass isolation window is specified in the data acquisition software and MS/MS spectra are acquired by selecting an optimized collisional energy for each of the targeted precursors. For example, in the first demonstration of nano-DESI MS/MS-MSI^34^ performed using a Q-Exactive Orbitrap instrument, the inclusion list contained 92 *m*/*z* windows and the isolation width was ±1 Da. Higher-energy collision-induced dissociation (HCD) spectra were acquired with an average acquisition rate of around 6.3 spectra/s, automatic gain control of 2 × 10^5^, and injection time (IT) of 100 ms. In MS/MS-MSI experiments, the pixel size is determined by the average acquisition time for the inclusion list. In this example, the average acquisition time for the inclusion list of 92 *m*/*z* values was ∼14.7 s and the scan speed was 10 μm/s. As a result, during the time when 92 MS/MS spectra were acquired generating data for one pixel, the sample moved by 147 μm under the nano-DESI probe. The spacing between the lines was 154 μm in that experiment, resulting an average pixel size of 147 μm × 154 μm. It was estimated that the targeted list of 92 *m*/*z* values contained more than 300 endogenous species that were simultaneously mapped in that study.^34^ The MS/MS acquisition mode enhances the sensitivity of nano-DESI MSI experiments. For example, in the study mentioned above, ornithine was only observed in MS/MS-MSI but was below the noise level in the full MS acquisition. Furthermore, the separation of closely spaced isobaric and some isomeric species has been demonstrated in that study. Figure 4a shows the MS/MS spectrum of *m*/*z* 808 ± 1, according to which two isobaric species, [PC 36:2 + Na]^+^ and [PC 38:5 + H]^+^, separated by 0.0024 Da contribute to the observed fragmentation. Figure 4b shows ion images of the fragments associated with these two isobaric species indicating their distinct localization across different regions of the tissue.

In another study, nano-DESI MS/MS-MSI was combined with an online photochemical derivatization approach for imaging of isomeric phospholipids.^36^ This study used an inexpensive light source and a photosensitizer (Rose Bengal) to promote photooxidation of unsaturated endogenous lipids to hydroperoxides. HCD of hydroperoxides generates diagnostic fragments that pinpoint the location of C=C bonds in different lipid species. MS/MS imaging of hydroperoxides revealed the spatial localization of positional lipid isomers in rat brain tissue, which cannot be achieved in MS mode.

Nano-DESI MSI coupled with a triple quadrupole (QqQ) mass spectrometer operated in the multiple reaction monitoring (MRM) acquisition mode has been used to distinguish between isobaric phospholipids in mouse brain tissue and isomeric eicosanoids in rat kidney tissue.^49^ Fast acquisition was achieved using a short dwell time of 5 ms for each MRM transition. Remarkably, isobaric species that require a mass resolving power of 3,800,000 in full MS mode were readily separated using a cost-effective mass analyzer with unit mass resolution. Furthermore, the sensitivity of MRM acquisition mode improves the limit of detection and quantification of biomolecules, which is particularly advantageous for imaging of low-abundance species such as eicosanoids in biological tissues.

### MS^3^ Mode

The capabilities of nano-DESI MS/MS-MSI have been further expanded by employing the MS^3^ imaging mode. This approach has been utilized to explore how the relative abundance of isomeric oleic acid (OA) and vaccenic acid (VA) varies across a tissue section through silver cationization of fatty acids (FAs) to generate diagnostic fragments in MS^3^ mode. Binding of silver to the C=C double bond in the monounsaturated FA has been used to pinpoint the position of the double bond and thereby differentiate the isomeric FAs.^35^ The method has been applied to mapping the distribution of %OA in different regions of a rat brain tissue.^35^ A similar approach has been used to differentiate and map the distribution of major prostaglandin isomers PGE2, PGD2, and Δ12-PGD2 in mouse uterine embryo implantation sites.^52^ In another study, nano-DESI MSI in MS^3^ mode has been combined with on-tissue chemical derivatization to enable imaging of phospholipid C=C and *sn-position* isomers in mouse brain tissues.^54^

### Ion Mobility Spectrometry Mode

**Figure 5:**
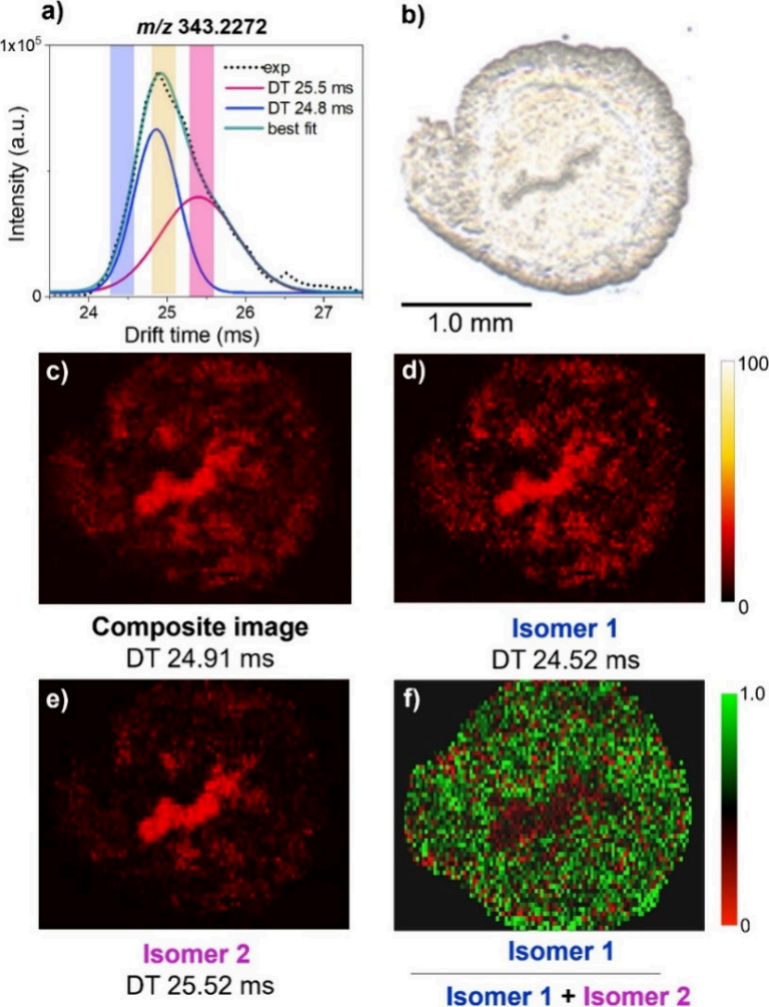
(a) Experimental and deconvoluted arrival time distribution (ATD) of *m*/*z* 343.2272. The experimental ATD (dotted line) is overlaid on top of the best fit profile (solid green line). ATD profiles of isomers 1 and 2 are shown as blue and pink lines, respectively. Colored bars indicate the region selected for image generation, with the yellow region being for the composite image. (b) Optical image of uterine tissue section. (c) Composite ion image generated at drift time (DT) 24.91 ms. (d) Ion image of isomer 1 generated at DT 24.52 ms. (e) Ion image of isomer 2 at DT 25.52 ms. (f) The fractional distribution image (FDI) shows that the relative abundance of isomer 1 is decreased in the luminal epithelium region. Reproduced with permission from ref (37). Copyright 2021 Elsevier.

Ion mobility spectrometry (IMS) is a gas phase separation technique which has been coupled with MSI (IMS-MSI) to enable the separation of isomeric and isobaric species and reduce spectral complexity.^55,56^ This approach significantly enhances the molecular coverage and specificity of MSI experiments. Both drift tube IMS (DTIMS) and trapped IMS (TIMS) have been coupled with nano-DESI MSI for imaging of biomolecules with a spatial resolution of better than 25 μm with isobaric and isomeric separation.^37,38^ For DTIMS MSI experiments, the ion accumulation time in the ion funnel trap is optimized to reduce the space charge effect, while the front ion funnel RF level is adjusted to minimize in-source fragmentation. Although DTIMS provides an accurate measurement of collision cross sections (CCS), which facilitates the identification of the observed analytes, the mobility resolution of commercial DTIMS systems (∼60) is often insufficient to resolve lipid isomers. In some cases, peak shape analysis may be used to distinguish the overlapping species. For example, a peak fitting approach was used to successfully resolve the spatial distributions of two isomers of two isomeric components at *m*/*z* 343.2272 (an oxidized FA) as illustrated in Figure 5.^37^

**Figure 6:**
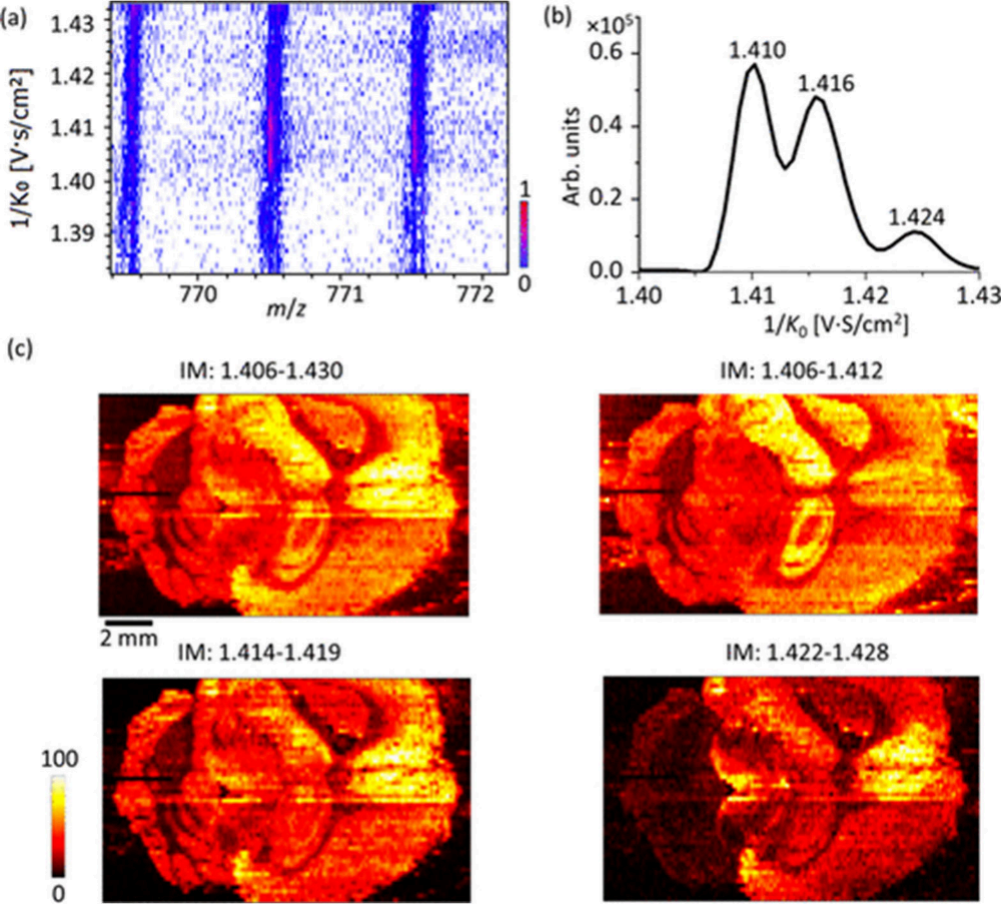
(a) A heatmap of a narrow *m*/*z* range of a mass spectrum averaged over a line scan. (b) Extracted ion mobilogram of *m*/*z* 770.51 ± 0.02. (c) Ion images of *m*/*z* 770.5077 extracted over four mobility ranges. Three narrow mobility scan windows of 1/*K*_0_ = 1.38–1.43, 1.42–1.47, and 1.46–1.51 were used in this study. Reproduced with permission from ref (38). Copyright 2023 American Chemical Society.

Higher mobility resolution of up to 300 for isomer separation in nano-DESI MSI may be achieved using TIMS.^38^ For example, nano-DESI MSI in a timsTOF system that been used to resolve three species at *m*/*z* 770.5077 as shown in Figure 6.^38^ Two of these species are isomers and the third one is a closely spaced isobar, which cannot be readily resolved on most commercial high-resolution MS systems. We note that this high mobility resolution is achieved over a limited ion mobility range. Therefore, multiple mobility windows were included in the experimental workflow to facilitate the detection of a broader range of species.

It is worth noting that for all targeted acquisition modes, such as MS/MS, MS^3^, IMS and SIM, the more windows are included in a method, the larger is the pixel size. Consequently, these acquisition modes often limit the achievable spatial resolution of the imaging experiment. To a certain extent, this challenge can be mitigated by employing a slower scan speed. Balancing the number of analysis windows and the quality of ion images ultimately rests with the user.

## Data Processing

To convert data acquired as multiple line scans into MS images, the intensities of desired peaks must be extracted from the data files and each mass spectrum must be spatially aligned. With the notable exception of Agilent QqQ data, MSConvert^57^ can convert MS data into the open-source. mzML format, which is accessible in Python,^58−60^ R,^57,60,61^ and other coding languages. To visualize an experiment using a targeted approach, a list of *m*/*z* values is generated, and the corresponding signal intensities are extracted from each mass spectrum. A simple intensity extraction method is to sum the intensity values for each *m*/*z* within given tolerance windows. The sampling time, indicating the time at which each mass spectrum was acquired, is used to determine the sampling location. In our experience, a combination of different delays during acquisition results in nonuniformly spaced mass spectra in nano-DESI experiments which necessitates the alignment of the data prior to visualization. An alignment procedure is typically performed by first normalizing the sampling times in each line to the same range, ex. [0,1]. Next, each line is resampled along uniformly distributed time points using nearest-neighbor interpolation. The resampled spectra are then combined to obtain a 3-axis image array (x-position, y-position, *m*/*z* value) of intensity values, which can be visualized as MS images. This image array can be normalized to the total ion current (TIC) to account for fluctuations in signal. Alternatively, it can be normalized to the signal of an internal standard that is present in the working solvent at a known concentration to account for matrix effects, which are described later in the text.

Several software packages including Ion-to-image (i2i),^62^ MSIgen (https://github.com/LabLaskin/MSIGen),^63^ MSIQuickView,^64^ and Mozaic MSI (Spectroswiss, Lausanne, Switzerland)^12^ have been developed or adapted to enable the visualization of nano-DESI MSI data. MSIQuickView is the first software specifically developed for nano-DESI MSI visualization and can process full MS data in the Thermo Fisher. raw format. It has been superseded by i2i which was developed in MATLAB and supports Thermo Fisher data containing MS^n^, MRM, and SIM data by converting the files into the open format. mzML using MSConvert.^57^ Additionally, i2i provides data analysis tools such as peak picking and ROI analysis in a user-friendly GUI. MSIGen is a Python package that supports the visualization of MS^1^, MRM, and MS^2^ with or without IMS data by conversion to. mzML format, or by directly using certain commercial formats. Furthermore, MSIGen provides a user-friendly GUI and other interface options, for accessibility and easy integration with user workflows. Another workflow has been developed for the visualization and analysis of IMS-MSI data acquired on Agilent IM-QTOF that relies on multiple programs^65^ and requires the user to modify it for compatibility before use.^66^ Mozaic MSI is a proprietary software requiring a purchased license for use. It supports common data formats including. mzML and commercial formats using multiple acquisition modes including MS^1^, MS^2^, MRM, and SIM. It contains options for MS and image processing, including peak picking, ROI analysis, and comparison of spatial distributions.

## Spatial Resolution

**Figure 7:**
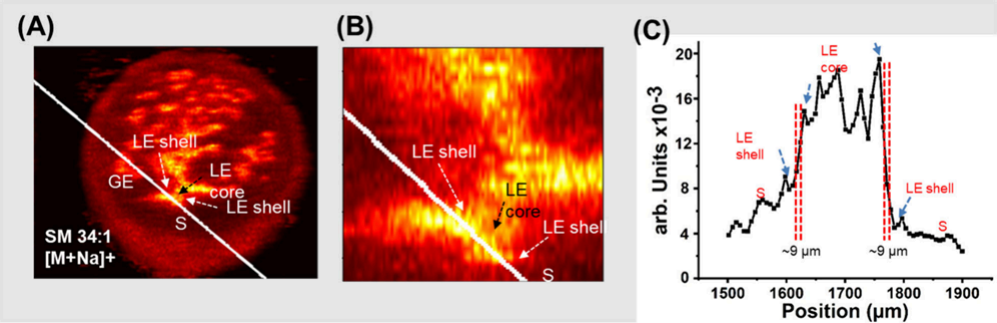
(A) Ion images of a mouse uterine tissue for the [M + Na]^+^ ion of SM 34:1 normalized to the TIC, showing the luminal epithelium (LE), glandular epithelial cells (GE) and the stroma surrounding it (S). (B) An expanded view of the region between LE and stroma regions. (C) Line profiles along the white line in the ion image for spatial resolution calculations. Red dashed lines highlight the regions over which the signal changes between 20% and 80% of the maximum intensity. Reproduced with permission from ref (48). Copyright 2023 Elsevier.

The spatial resolution of MSI is a critical characteristic that determines the level of detail and the ability to resolve closely spaced molecular features in a sample. The spatial resolution typically defines the smallest distinguishable feature size in the acquired image. In nano-DESI MSI, the spatial resolution is usually determined by the size of the liquid bridge on the sample surface. However, when the distance that the sample travels between the successive mass spectral acquisitions is larger than the size of the liquid bridge, the acquisition rate of a mass spectrometer may limit the spatial resolution. As in several other techniques that do not use a square grid for data acquisition, the upper limit of the spatial resolution in nano-DESI MSI is estimated by measuring the distance across which the signal intensity of a specific *m*/*z* changes from 20% to 80% of its maximum value. This method can only be applied to *m*/*z* features that exhibit steep concentration gradients in the sample.^15,67^ An example is illustrated in Figure 7 where the signal intensity gradient for a [M + Na]^+^ ion of SM 34:1 across the sharpest feature in the mouse uterine tissue was used to determine a spatial resolution of 9 μm.^48^

Because in nano-DESI MSI the stage controlling the position of the sample moves at a constant speed in the *X*-direction and subsequently steps in equal intervals in the *Y*-direction, the pixel width (pixel size along the *X*-axis) is dependent on the scan speed, and the pixel height is determined by the spacing between the lines used in the imaging experiment. To obtain high spatial resolution, it is important to ensure that the pixel width is smaller than the size of the liquid bridge on the surface and reduce the spacing between the lines. The size of the liquid bridge is controlled by the design of the nano-DESI probe. In capillary-based probes (Figures 1a and 1b), the diameters of both capillaries, their position relative to each other and instrument inlet, and the flow rate of the working solvent determine the size of the liquid bridge and thus the sampling area. The properties of the solvent and the composition of the sample influence surface wetting, which may affect the size of the liquid bridge. If the tissue is hydrophilic, solvent spreading may result in a bigger liquid bridge, particularly with more polar solvents. The size of the liquid bridge may be reduced by adding a nonpolar component or water to increase surface tension. As discussed later, changes in solvent composition may alter the extraction efficiency of some classes of analytes. Therefore, the optimization of the solvent composition may be challenging.

**Figure 8:**
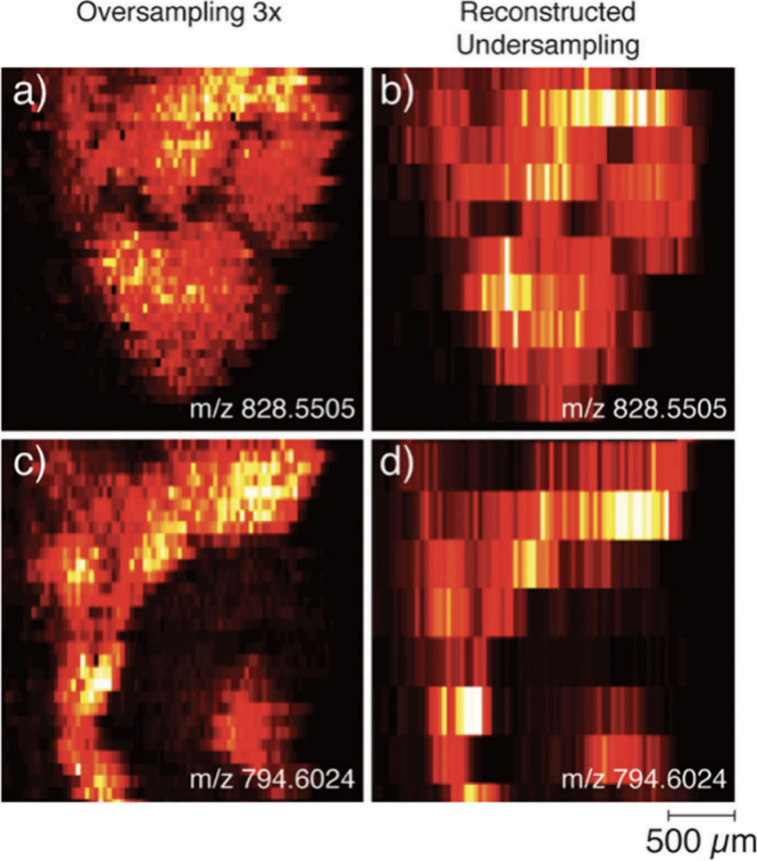
Demonstration of the use of oversampling to improve the spatial resolution in nano-DESI MSI. Oversampling and in silico reconstructed undersampling displaying the improvement in the spatial resolution for oversampling of partial spinal cord ion images with nano-DESI MSI. (a) and (c) show the ion image of PC 38:6 (*m*/*z* 828.5505 [M + Na]^+^) and PC 36:1 (*m*/*z* 796.5823 [M + Na]^+^), respectively. Analogous reconstructed undersampling ion images are shown in (b) and (d). The ion images show relative intensities from dark to bright, individually scaled, and are all proportional in size to the scale bar in the lower right corner of the figure. Reproduced with permission from ref (67). Copyright 2018 American Chemical Society.

The spatial resolution can also be enhanced using oversampling as illustrated in Figure 8.^67^ In this approach, the spacing between the lines is smaller than the size of the liquid bridge. As a result, in each line scan, the probe resamples part of the previously analyzed tissue. Analyte depletion due to repeated sampling effectively reduces the scanning area, allowing for finer features to be resolved. This approach was first validated for nano-DESI MSI by Duncan et al.^67^ as shown in Figure 8. Due to the higher spatial resolution from oversampling, the low intensity signals from morphological regions could be distinguished from noise, and many features that were not obvious inion images generated using the conventional data acquisition approach were more clearly revealed with oversampling. Furthermore, it has been demonstrated that oversampling does not cause analyte redistribution in the sample.^67^

## Throughput

The utility of MSI for 3D imaging and other time sensitive applications is strongly dependent on the experimental throughput. Nano-DESI does not require any special sample preparation. Therefore, the experimental throughput is determined by the scan speed, the spatial resolution, and the area of the sample.^68^ The scan speed is optimized to maintain a stable liquid bridge on the sample surface, ensure efficient transfer of the desorbed analytes to the inlet through the nanospray capillary, and match the acquisition rate of the mass spectrometer. For capillary-based probes, typical scan speed is in the range of 5–50 μm/s. Meanwhile, MFPs are more robust and can be operated at much higher scan speed of 100–500 μm/s.^43,46,48^ In order to match the acquisition rate of a mass spectrometer, high resolution experiments are often performed using slower scan speed. In addition, the spacing between the lines is decreased and the number of lines is increased thereby increasing the acquisition time. For example, imaging of an area of 10 × 10 mm^2^ with an acquisition rate of 10 Hz and a spatial resolution of 10 μm would require a total acquisition time of ∼28 h. Meanwhile, imaging of the same area with a 100 μm resolution would take ∼17 min. The optimal balance between throughput and spatial resolution depends on the specific requirements of the application.

Advances in MSI technology, such as faster scanning and improved data processing algorithms, help mitigate the trade-off between the throughput and spatial resolution of nano-DESI MSI experiments.^68^ For example, MFPs have been successfully used to achieve a 10-fold improvement in the experimental throughput without a substantial loss in sensitivity or image quality, which enabled imaging of large tissue sections with at 10 μm spatial resolution in a reasonable time.^48^ Another approach to improving the throughput of MSI experiments relies on decreasing the number of sampling locations using computational approaches such as sparse sampling.^69^ The recently developed deep learning approach for dynamic sparse sampling (DLADS) uses a metric called estimated reduction in distortion to predict information-rich sampling locations and reconstruct high quality images based on the sparely sampled data.^70^ DLADS has been used to achieve a 2–3 fold improvement in the experimental throughput of nano-DESI MSI and is predicted to provide a 10-fold improvement for MSI experiments involving point-wise acquisition such as MALDI.^70^

## Solvent Selection

One of the major advantages of nano-DESI is the versatility in solvent selection. Solvent components can be selected to enhance the extraction and ionization efficiency of targeted classes of biomolecules. Conventional nano-DESI MSI experiments use 9:1 Methanol/H_2_O (v/v) solvent, which provides efficient extraction and ionization of phospholipids, mono- and diacylglycerols, and some metabolites. To enhance the extraction of triacylglycerides, a hydrophobic component has been incorporated into the nano-DESI solvent. Both MeOH:DCM 6:4 (v/v) and MeOH:ACN:Tol 5:3.5:1.5 (v/v/v) are well-suited for imaging of triacylglycerides.^10^ Meanwhile, imaging of N-glycans is best performed using a higher water content (MeOH:H_2_O 70:30 (v/v)) in the solvent.^14^

For imaging of proteoforms, both ACN/H_2_O/CH_3_COOH (65/34/1, v/v/v) and ACN/H_2_O/HCOOH (80/20/0.1, v/v/v) were used as extraction solvents and the tissue was delipidated using several ethanol washes and a wash in CH_3_Cl.^15,17^ The use of an acidified solution further aids in the solubility and ionization of proteins. A similar extraction solvent composed of ACN/H_2_O/CH_3_COOH 60/39.4/0.6 (v/v/v) was used for nano-DESI proteoform imaging by individual ion mass spectrometry.^16^ For imaging of native proteins and protein complexes, a solvent system composed of 200 mM ammonium acetate (at pH ∼ 7) and 0.125–0.5% C_8_E_4_ detergent has been used.^18,19,71,72^

Ionic dopants have been utilized to promote the ionization of targeted classes of biomolecules. For example, Weigand et al. incorporated 500 μM NH_4_F into the traditional 9:1 Methanol/H_2_O (v/v) solvent and reported a substantial signal enhancement of lipids in negative ionization mode.^73^ Increased signals were observed for multiple lipid classes including fatty acids, lysophosphatidylethanolamines (LPEs), phosphatidylethanolamines (PEs), phosphatidylinositols (PIs), phosphatidylserines (PSs), and prostaglandins (PGs). As mentioned earlier silver adduction that enhances the ionization efficiency of steroids,^11^ fatty acids,^35^ phospholipids,^39^ and prostaglandins^52,74^ has been used for imaging and differentiation of positional isomers of these important classes of biomolecules. In addition to biomolecules, elemental MSI of endogenous sodium and potassium has been implemented through in situ host–guest chemistry using crown ethers.^75^

**Figure 9:**
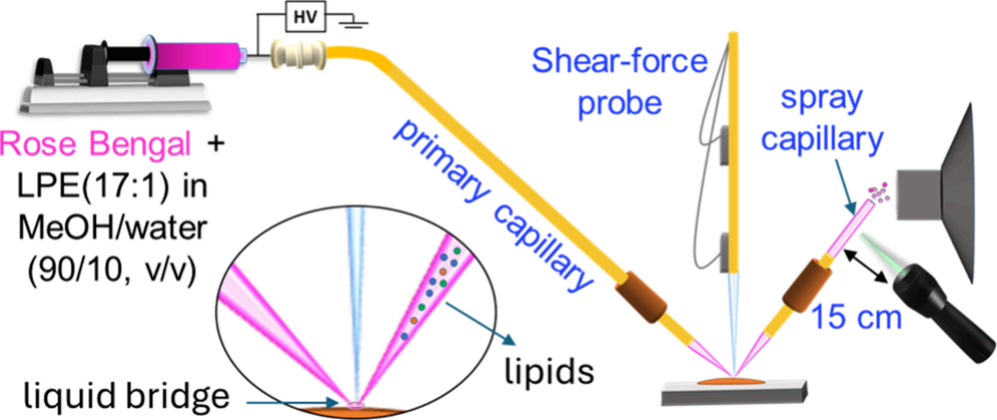
Coupling of nano-DESI to online photochemical reactions through the incorporation of Rose Bengal, a photosensitizer, in the working solvent and a laser focused on the nanospray capillary for the online conversion of extracted unsaturated lipids to lipid hydroperoxides. Adapted with permission from ref (36). Copyright 2021 Wiley-VCH GmbH.

Online derivatization of the extracted analytes can be readily achieved in nano-DESI MSI experiments by adding a reagent to the solvent. After their extraction, analytes travel through the nanospray capillary to the MS inlet for ∼1 s. This offers an opportunity to couple nano-DESI MSI with fast, online postextraction derivatization of endogenous analytes. For example, online chemical derivatization of phosphatidylcholine species has been achieved in nano-DESI MSI by adding Girard T reagent to the solvent.^10^ Furthermore, through the incorporation of a photosensitizer into the solvent and using a laser focused on the tip of the nanospray capillary as shown in Figure 9, Unsihuay et al. performed online photochemical derivatization of unsaturated lipids for positional isomer identification and isomer-selective imaging.^36^

## Matrix Effects

**Figure 10:**
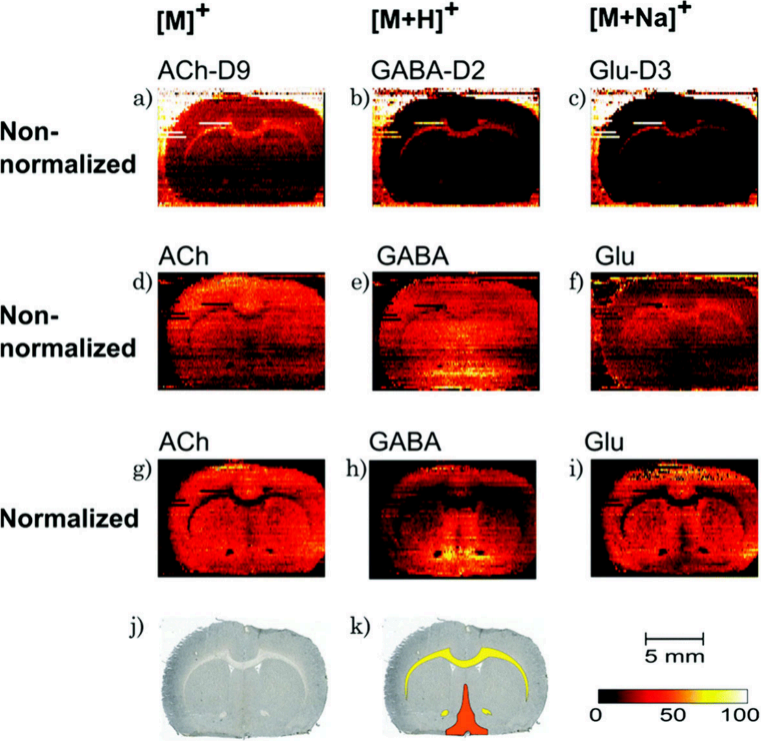
(a)-(c). Ion images of deuterated internal standards of acetylcholine, GABA, and glutamate. (d)-(f). Raw ion images of each neurotransmitter. (g)-(i). Ion images of each analyte when normalized to its respective internal standard, revealing different localizations than originally presented without normalization, and thereby removing matrix effects. (j). Optical image of rat brain tissue. (k). Optical image of analyzed brain tissue section with white matter regions highlighted in yellow and the medial septum-diagonal band complex highlighted in orange. Reproduced with permission from ref (31). Copyright 2016 The Royal Society of Chemistry.

In MSI experiments, all the extracted molecules from one pixel are ionized simultaneously and therefore compete for charge. Therefore, peak intensities produced in a mass spectrum are determined by analyte concentrations, their ionization efficiencies, and the ability to compete for charge with hundreds of other molecules extracted from the same location on a tissue.^76^ Signal suppressions in ionization resulting from this competition, also called “matrix effects” are commonly observed in MSI experiments.^76^ Matrix effects are detrimental to the accurate measurement of concentration gradients in the sample.^77^ Several approaches have been developed to compensate for matrix effects.^76,78−80^ Solvent versatility in nano-DESI allows for internal standards to be easily incorporated into the solvent for analyte normalization. When choosing internal standards, it is imperative to choose molecules of similar structure to analytes of interest to ensure similar ionization/suppression. In Figure 10, signals of neurotransmitters of interest including acetylcholine, GABA, and glutamate observed in nano-DESI MSI of mouse brain tissues were normalized to signals of their respective deuterated standards.^31^ This figure demonstrates a stark difference in the localizations of the analytes observed without and with normalization. These differences are attributed to the pronounced changes in the chemical composition of lipids between white and gray matter of the brain tissue, resulting in distinct matrix effects analytes experience when extracted from different regions of the tissue. In a similar study, matrix effects of PC lipids of different acyl chain compositions were explored, and it was concluded that molecular size and structure are two key factors that determine ionization efficiency and on-tissue signal suppression.^30^ In addition, variations of alkali metal concentrations across the tissue affects the formation of alkali metal adducts typically observed in positive mode nano-DESI MSI experiments.^77^ It has been demonstrated that matrix effects can be effectively compensated for by normalizing the signals of endogenous analytes to the signals of the same adducts of their respective internal standards.^77^ For lipids, only one standard per lipid class can be used to compensate for matrix effects.^30^ Meanwhile, deuterated standards are commonly used for accounting for matrix effects in imaging of metabolites.^22^

## Conclusions

Nano-DESI MSI offers unique capabilities in terms of spatial resolution, throughput, quantification, and molecular coverage rendering it an attractive technique of significant interest to the MSI community. In this tutorial, we describe the operational principles of nano-DESI and discuss critical factors and practical aspects that must be considered when using this technique. We outline data acquisition and analysis procedures, emphasizing the essential parameters affecting the spatial resolution and experimental throughput. Additionally, we describe important considerations in selecting the working solvent for nano-DESI MSI experiments. We anticipate that this tutorial will provide readers with a deep understanding of nano-DESI for future exploration and applications.
